# Inexpensive synthetic-based matrix for both conventional and rapid purification of protein A- and tandem affinity purification-tagged proteins

**DOI:** 10.1016/j.ab.2009.09.045

**Published:** 2010-02-15

**Authors:** Kenneth E. Sawin, Claudia C. Bicho, Hilary A. Snaith

**Affiliations:** Wellcome Trust Centre for Cell Biology, University of Edinburgh, Edinburgh EH9 3JR, UK

## Abstract

Immunoglobulin G (IgG)–Sepharose is often used for purification of protein A- and tandem affinity purification (TAP)-tagged proteins from eukaryotic cells, but because it is based on an agarose matrix, it is not always optimal for all proteins. Synthetic matrices such as IgG–Dynabeads have improved properties over IgG–Sepharose but are generally expensive. Here we describe the preparation and properties of an IgG matrix based on Fractogel EMD beads. As a synthetic-based matrix, IgG–Fractogel has clear advantages over IgG–Sepharose. IgG–Fractogel can also be used in applications that usually use IgG–Dynabeads but at a significantly reduced cost.

Affinity tags provide a powerful method to purify proteins for both analytical and preparative purposes. One particularly important advance in purifying endogenously expressed eukaryotic proteins has been the tandem affinity purification (TAP)^1^ approach [1,2] in which two different affinity tags are used in succession to purify a doubly tagged protein. These and related methods often use one or more repeats of a synthetic immunoglobulin-binding domain from *Staphylococcus aureus* protein A (Z domain [3]). Protein fusions to two Z domains (ZZ) are most often used and bind to immunoglobulin G (IgG) under a range of conditions.

IgG–Sepharose is a widely used and inexpensive matrix for purification of proteins fused to Z domains. Purification of native proteins and protein complexes typically involves a cleavage step by tobacco etch virus (TEV) or rhinovirus 3C protease via the introduction of an artificial cleavage site between the tag and the protein of interest. Protease digestion releases the protein while leaving the tag bound to the matrix. In ongoing work to identify protein interactors copurifying with the fission yeast cell polarity regulator Tea1 [4], we found that C-terminally TAP-tagged Tea1 (in which ZZ was one of the affinity tags) was able to bind to IgG–Sepharose and the tag was readily cleaved from Tea1 by TEV protease, as expected. However, unexpectedly, the cleaved Tea1 protein was not released from IgG–Sepharose under native conditions (Fig. 1A). This was particularly puzzling because untagged Tea1 itself did not bind to IgG–Sepharose (data not shown). Although the cleaved Tea1 protein could be released from IgG–Sepharose with mild chaotropes or denaturing conditions, this made it impossible to continue to a second purification step without the loss of potentially important interacting proteins. IgG can be coupled to epoxy-derivatized Dynabeads (Invitrogen), and this has been particularly effective for rapid single-step purification of tagged proteins [5]. We found that Tea1–TAP bound to IgG–Dynabeads was readily released by TEV cleavage (not shown). However, IgG–Dynabeads have a relatively low IgG-binding capacity and are expensive, making them less desirable for large-scale use. Therefore, we developed an alternative matrix for purification based on IgG coupling to epoxy-derivatized Fractogel EMD (Merck), a methacrylate-based “tentacle” resin in which functional groups are linked to beads by linear polymer chains. Overall, we have found that IgG–Fractogel combines the relative low cost of IgG–Sepharose with the ease of use and reduced “stickiness” of IgG–Dynabeads.

We coupled IgG to Fractogel EMD Epoxy using a modification of methods of Oeffinger and coworkers [5]. Compared with other coupling conditions, this provided a good yield in protein purifications without excessive nonspecific binding (data not shown). One significant modification was that, because Fractogel EMD Epoxy has a very high density of reactive groups (0.5–1.0 mmol/g), we partially deactivated the beads prior to coupling so as to avoid overcoupling individual IgG molecules to the resin. We also measured the extent of coupling directly on the beads by bicinchoninic assay (BCA) assay [6], which generates a soluble product even with immobilized protein [7]. A typical value for coupling is 1.5–2.0 mg of covalently bound IgG per milliliter of packed beads. Detailed protocols for coupling and protein assay are given in the supplementary material.

IgG–Fractogel can, in principle, be used in essentially any application where IgG–Sepharose is used. In our own experiments, we found that Tea1–TAP was quantitatively released from IgG–Fractogel after treatment with TEV protease (Fig. 1B), indicating a clear advantage of IgG–Fractogel over IgG–Sepharose for two-step purifications in this instance. We also compared the performance of IgG–Fractogel with that of IgG–Dynabeads in rapid single-step purifications (i.e., without TEV cleavage) following the protocols of Oeffinger and coworkers [5]. For this, we initially used lysates from cells in which a TAP tag was fused to the C terminus of Mto1, a low-abundance fission yeast protein involved in microtubule nucleation (also known as Mod20/Mbo1 [8,9]). After a brief incubation with cell lysates, beads were washed and the (uncleaved) protein was eluted from the beads [5]. Recovery of beads during washes used either a magnet for IgG–Dynabeads or a disposable column (Sigma C2353) for IgG–Fractogel. Because of the density of Fractogel EMD, the IgG–Fractogel beads settle very quickly during washes, and their opacity makes them extremely easy to observe and recover from the column. Using amounts of beads that were individually optimized for each of the two matrices, we found that IgG–Fractogel could be used to isolate Mto1–TAP with yield and purity approaching those of IgG-Dynabeads (Fig. 2A) but with a 10- to 20-fold reduced cost, taking into consideration both the cost of IgG and, more significant, the cost of the matrix. IgG–Fractogel appeared to have slightly higher nonspecific binding than IgG–Dynabeads, but it might be possible to improve this by reducing the amount of IgG coupled to the beads, given that the Fractogel matrix itself (i.e., without coupled IgG) shows very low protein binding (Fig. 2B). To test the usefulness of IgG–Fractogel more generally, we also compared IgG–Fractogel with IgG–Dynabeads in a single-step purification of a multiprotein complex, the budding yeast anaphase-promoting complex (APC) [10]**,** using a strain in which a TAP tag was fused to the C terminus of the APC component Apc4 [11]. Similar bands were pulled down with both IgG–Dynabeads and IgG–Fractogel (Fig. 2C), demonstrating the potential value of IgG–Fractogel for purification of multiprotein complexes.

In our single-step purifications, we noticed that some protein bands that were present in pull-downs using untagged cell extracts, and thus were indicative of nonspecific binding, were nevertheless increased in intensity in pull-downs using tagged cell extracts (Fig. 2A, “NS”). This was observed both with IgG–Dynabeads and with IgG–Fractogel. Although the basis for this behavior is not yet clear, it suggests that when these methods are used to identify proteins that copurify with a protein of interest, comparisons of what is isolated from untagged versus tagged cell extracts have the potential to give false-positive identifications and, thus, may be misleading if viewed too narrowly. One solution to this problem is to use a tagged but irrelevant cell extract, rather than an untagged cell extract, as a negative control. Depending on the methods of analysis available, other more sophisticated solutions using stable isotope labeling are also possible [12].

In conclusion, we have found that purifications on IgG–Fractogel can be performed at a significantly reduced cost relative to IgG–Dynabeads. The methods described here may be particularly useful when larger amounts of material are needed, such as for preparative biochemistry.

## Figures and Tables

**Fig. 1:**
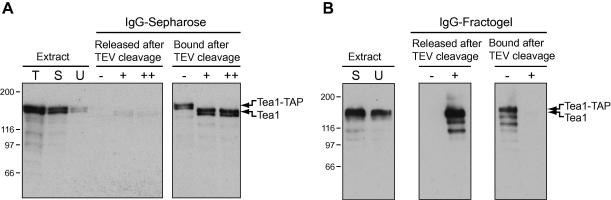
TAP-tagged Tea1 bound to IgG–Sepharose is cleaved by TEV protease but not released from beads, whereas TAP-tagged Tea1 bound to IgG–Fractogel is cleaved and released. (A) Anti-Tea1 Western blots showing Tea1–TAP in total fission yeast extracts (T), extract supernatants before incubation with IgG–Sepharose beads (S), and the fraction unbound to IgG–Sepharose (U). Remaining lanes show the Tea1 released or remaining bound to IgG–Sepharose after incubation without TEV protease (−) and with two different concentrations of TEV protease (+ and ++). (B) Anti-Tea1 Western blots showing Tea1–TAP in extract supernatants before incubation with IgG–Fractogel (S), the fraction unbound (U), and the amounts of Tea1 released or remaining bound to IgG–Fractogel after incubation without TEV protease (−) and with TEV protease (+). Molecular weight markers (in kDa) are shown at the left. Further details are provided in the supplementary material.

**Fig. 2:**
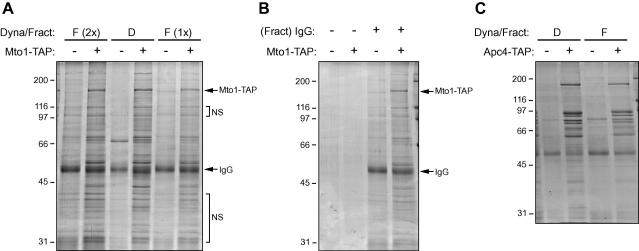
IgG–Fractogel recovers TAP-tagged proteins and complexes with yield and purity similar to IgG–Dynabeads. (A) Coomassie-stained sodium dodecyl sulfate–polyacrylamide gel electrophoresis (SDS–PAGE) showing pull-downs of extracts from wild-type controls (−) and Mto1–TAP-expressing fission yeast cells (+) using either IgG–Dynabeads (D) or two different amounts of IgG–Fractogel [F (2×) and F (1×)]. “NS” indicates representative protein bands that appear to be nonspecifically associated with both IgG–Dynabeads and IgG–Fractogel but, nevertheless, increase in intensity in pull-downs from tagged strains. (B) Coomassie-stained SDS–PAGE showing pull-downs of extracts from wild-type controls (−) and Mto1–TAP-expressing fission yeast cells (+) using either Fractogel that was not coupled to protein (−) or IgG–Fractogel (+). (C) Coomassie-stained SDS–PAGE showing pull-downs of anaphase-promoting complex (APC) proteins in extracts from wild-type controls (−) and Apc4–TAP-expressing budding yeast cells (+) using either IgG–Dynabeads (D) or IgG–Fractogel (F). IgG–Dynabeads and IgG–Fractogel pull down the same bands. APC components with molecular weight less than 30 kDa were run off the bottom of the gel. Molecular weight markers (in kDa) are shown at the left. Further details are provided in the supplementary material.

## Appendix A Supplementary data

Supplementary material 1 Supplementary text for Sawin et al.

Supplementary material 2 Coupling rabbit IgG to Fractogel EMD Epoxy.

## Footnotes

1 *Abbreviations used:* TAP, tandem affinity purification; IgG, immunoglobulin G; TEV, tobacco etch virus; BCA bicinchoninic acid.
