# Supplementary material for: Inexpensive synthetic-based matrix for both conventional and rapid purification of protein A- and tandem affinity purification-tagged proteins
Source: Anal Biochem. 2010 Feb 15;397(2):241–3. doi: 10.1016/j.ab.2009.09.045 (PMC2810510; doi:10.1016/j.ab.2009.09.045)
Supplement: Supplementary material 1 — Supplementary text for Sawin et al. [file mmc1.pdf]

Supplementary Text for Sawin *et al.*

Purification of TAP-tagged Tea1 on IgG-Sepharose and IgG-Fractogel

For purification on IgG-Sepharose in the experiment shown in Fig. 1A, cells expressing Tea1-TAP (C-terminal TAP tag, consisting of calmodulin-binding peptide (CBP), TEV cleavage site, and ZZ domain; [1]) from the endogenous *tea1*+ chromosomal locus (strain KS2719) were grown in 1 l 4xYES medium at 32° C and harvested in exponential phase at OD_{595} 4. The cell pellet was washed in 400 ml ice-cold 20 mM K HEPES, pH 7.5, resuspended in a final volume of 2.5 ml in the same buffer and snap frozen as pellets in liquid nitrogen. 4.5 g of frozen cell pellets were ground in liquid nitrogen using a Retsch RM100 electric mortar-grinder to 70% cell breakage. The resulting frozen cell powder was resuspended in 8 ml ice cold lysis buffer containing 20 mM K HEPES, pH 7.5, 100 mM KCl, 1.5 mM $MgCl_2$, 2 mM EDTA, 0.1% octyl-glucopyranoside, 1 mM DTT, and protease inhibitors 1 mM PMSF, 2 mM benzamidine, 20 μg/ml chymostatin, 20 μg/ml leupeptin, 20 μg/ml antipain, 20 μg/ml pepstatin, and 20 μg/ml E64. The extract was incubated at 4 °C for 15 min, centrifuged at 25000 x g for 20 min, and the supernatant incubated with 400 μl of packed IgG sepharose beads for 3 h at 4°C. The beads were washed three times with 10 ml lysis buffer, once with 10 ml lysis buffer plus 200 mM KCl, and once more with lysis buffer. The beads were resuspended in 4.5 ml lysis buffer, giving 10 mg of starting cells per μl of packed beads. 17 μl of beads were incubated with buffer only, 10 units AcTEV (Invitrogen) or 20 units AcTEV for 2 h at 4°C, after which the supernatant was removed and 2x SDS PAGE sample buffer added to resulting supernatant and beads fractions. All samples were boiled for 5 min, run on 8% SDS PAGE, transferred to nitrocellulose membrane and probed with anti-Tea1 antibodies [2] and chemiluminescent detection to assess efficiency of TEV cleavage and release of Tea1 from the beads.

For purification on IgG-Fractogel in the experiment shown in Fig. 1B, cells expressing a variant Tea1-TAP (C-terminal TAP tag, consisting of S peptide from RNAse A, TEV cleavage site, and ZZ domain; [3]; strain KS2739) from the endogenous *tea1*+ chromosomal locus were grown in 1.5 l 4xYES at 32° C and harvested in exponential phase at OD_{595} 8. The cell pellet was washed in 400 ml ice-cold 25 mM sodium phosphate pH 8.0, 60mM β-glycerophosphate, 15 mM *para*-nitrophenylphosphate, 0.1 mM sodium orthovanadate, 1 mM sodium azide and 50 mM sodium fluoride, resuspended in a final volume of 5.5 ml in the same buffer and snap frozen as pellets in liquid nitrogen. 2 g of cells were ground in liquid nitrogen using the Retsch grinder. The resulting frozen cell powder was resuspended in 4 ml ice cold lysis buffer containing 25 mM sodium phosphate pH 8.0, 150 mM sodium chloride, 2 mM EDTA, 1% Igepal CA-630, 1 mM DTT, phosphatase inhibitors 60 mM β-glycerophosphate, 15mM *para*-nitrophenylphosphate, 0.1 mM sodium orthovanadate, 1 mM sodium azide and 50 mM sodium fluoride, and protease inhibitors 2 mM PMSF, 2 mM benzamidine, 20 μg/ml chymostatin, 20 μg/ml leupeptin, 20 μg/ml antipain, 20 μg/ml pepstatin, and 20 μg/ml E64. The extract was incubated at 4°C for 30 min, centrifuged at 2000 x g for 2 min, and the supernatant was then centrifuged at 50000 x g for 30 min. 4 ml of this supernatant was incubated with 40μl of packed IgG-Fractogel beads for 2h at 4°C. The beads were washed five times with 10 ml of wash buffer (25mM Tris-HCl pH 8.0, 150 mM sodium chloride, 0.1% Igepal CA-630 and 1mM DTT). The beads were resuspended in 1 ml of wash buffer plus 0.5 mM EDTA, and were incubated with buffer only or 30 units of home-prepared TEV at 4ºC for 2h, after which the supernatant was removed and 2x SDS

PAGE sample buffer added to resulting supernatant and bead fractions. All samples were boiled for 5 min, run on 8% SDS PAGE, transferred to nitrocellulose membrane and probed with anti-Tea1 antibodies to assess efficiency of TEV cleavage and release of Tea1 from the beads.

We note that although there are differences between the strains and methods of extract preparation in the two specific examples shown in Fig. 1, further experiments indicate that these did not effect the differential release of Tea1 from the two different types of beads.

Preparation of IgG-matrices

IgG-Fractogel was prepared as described in the accompanying protocol. Fractogel without IgG (Fig. 2B) was prepared using the same partial deactivation of reactive groups used in preparation of IgG-Fractogel, and the beads were kept stored in dH2O at 4°C. IgG-Dynabeads were prepared as described by Oeffinger *et al.* [4].

Single-step purification of TAP-tagged proteins on IgG Fractogel and IgG-Dynabeads

For the experiments shown in Fig. 2A, wild-type cells (strain KS515) and cells expressing Mto1-TAP (C-terminal TAP tag, consisting of RNAse A S-peptide, TEV cleaveage site, and ZZ domain; [3]) from the endogenous *mto1*+ chromosomal locus (strain KS3524) were grown in 4xYES at 30° C and harvested in exponential phase at OD_{595} 15. The cell pellet was washed twice in 1/6 culture volume ice-cold 10 mM sodium phosphate pH 7.5, 0.5 mM EDTA, resuspended in a final volume of 0.3 ml of the same buffer per g of wet cell mass and snap frozen as pellets in liquid nitrogen. Cells were ground in liquid nitrogen using the Retsch grinder. The resulting frozen cell powder was resuspended in 2 ml ice cold extract buffer per 1 g of frozen cell powder. Extract buffer was 10 mM sodium phosphate pH 7.5, 100 mM sodium chloride, 0.5 mM magnesium chloride, 1.5 mM EGTA, and 5% glycerol, with protease inhibitors 1 mM PMSF, 1mM AEBSF, 1 mM benzamidine, 5 μg/ml chymostatin, 5 μg/ml leupeptin, 5 μg/ml antipain, 5 μg/ml pepstatin, and 5 μg/ml E64. Triton X-100 was added to 1% final (v/v) and the extract was briefly centrifuged twice at 3,200 x g for 5 min to remove unbroken cells and large debris.

Purification methods were modelled on the methods of Oeffinger *et al.* [4]. To compare behavior of IgG-Dynabeads vs. IgG-Fractogel, 2 ml of extract from either wild-type or Mto1-TAP-expressing cells was mixed with either 1.5×10^8 IgG-Dynabeads or 25 (=1X) or 50 (=2x) μl of packed IgG-Fractogel beads and incubated with gentle mixing at 4°C for 45 min. Beads were then washed quickly using either a magnet (Dynabeads) or by gravity flow in a disposable column (Sigma C2353; Fractogel). Washes consisted of 2 x 4 ml extract buffer containing 1% TX-100 (without protease inhibitors), 2 x 4ml extract buffer containing 0.1% TX-100 (without protease inhibitors), and 1 x 4 ml of ammonium acetate pre-elution buffer (0.1 M ammonium acetate, 0.1 mM magnesium chloride, 0.2% Tween-20). Washes used a "jet" of liquid from a Gilson Pipetman P5000, to resuspend beads. Beads were then collected and washed in 1.2 ml of ammonium acetate pre-elution buffer, and eluted twice for 20 min each with 0.5 ml of 0.5 M ammonium hydroxide, 0.5 mM EDTA. The two elutions were combined, lyophilized, resuspended in SDS-PAGE sample buffer and run on 10% SDS-PAGE.

To compare protein-binding to IgG-Fractogel vs. Fractogel without IgG (Fig. 2B), 2 ml of extract from either wild-type or Mto1-TAP strains was mixed with either 25 μl packed beads of IgG-Fractogel or Fractogel without IgG and incubated with

gentle mixing at 4°C for 30 min. Beads were then washed and further processed as described immediately above.

Purification of the budding yeast APC (Fig. 2C) was done essentially as described above for fission yeast Mto1, except that strains JB811 (untagged negative-control strain) and SJ148.2 Apc4-SZZ tub2-401 (tagged APC strain) were used (both were the kind gift of Dr. Kevin Hardwick, University of Edinburgh). Either 1.5 x 10^8 IgG-Dynabeads or 25 µl of packed IgG-Fractogel beads were used in the pull-downs.

References

[1] J.J. Tasto, R.H. Carnahan, W.H. McDonald, and K.L. Gould, Vectors and gene targeting modules for tandem affinity purification in Schizosaccharomyces pombe. Yeast 18 (2001) 657-62.
[2] H.A. Snaith, I. Samejima, and K.E. Sawin, Multistep and multimode cortical anchoring of tea1p at cell tips in fission yeast. EMBO J. 24 (2005) 3690-9.
[3] C. Brune, S.E. Munchel, N. Fischer, A.V. Podtelejnikov, and K. Weis, Yeast poly(A)-binding protein Pab1 shuttles between the nucleus and the cytoplasm and functions in mRNA export. RNA 11 (2005) 517-31.
[4] M. Oeffinger, K.E. Wei, R. Rogers, J.A. DeGrasse, B.T. Chait, J.D. Aitchison, and M.P. Rout, Comprehensive analysis of diverse ribonucleoprotein complexes. Nat Methods 4 (2007) 951-6.
