# Supplementary material for: Inexpensive synthetic-based matrix for both conventional and rapid purification of protein A- and tandem affinity purification-tagged proteins
Source: Anal Biochem. 2010 Feb 15;397(2):241–3. doi: 10.1016/j.ab.2009.09.045 (PMC2810510; doi:10.1016/j.ab.2009.09.045)
Supplement: Supplementary material 2 — Coupling rabbit IgG to Fractogel EMD Epoxy. [file mmc2.pdf]

Coupling rabbit IgG to Fractogel EMD Epoxy

Kenneth Sawin
Wellcome Trust Centre for Cell Biology
University of Edinburgh, UK

Last modified 15 September 2009

Note: All manipulations and reactions are done at room temperature unless otherwise stated, in disposable conical polypropylene tubes (15 or 50 ml, as needed). The following protocol is for processing 1 g of Fractogel EMD Epoxy. It can be scaled-up as needed.

Materials:

- Fractogel EMD Epoxy (size 40-90 μm, Merck KGaA, Darmstadt, Germany, catalog number 1.16961.0010, available in the UK through VWR)
- Rabbit IgG (Sigma, catalog number I5006)
- 0.5 M sodium phosphate pH 7.5 stock solution (dilute as needed)
- 3 M ammonium sulfate solution
- PBS (phosphate-buffered saline) solution
- 100 mM glycine HCl, pH 2.5
- 10 mM Tris pH 8.8
- 100 mM Triethylamine (prepare fresh)
- 20% Triton X-100 stock solution
- 10% sodium azide stock solution
- Laemmli SDS-PAGE sample buffer without bromophenol blue or reducing agents
- BCA (bicinchoninic acid) protein assay reagent

- 15 and 50 ml disposable conical propylene tubes
- Disposable plastic column (Sigma, catalog number C2353)
- 0.22 μm syringe filter and 10 or 20 ml syringe
- General lab plasticware

Partial deactivation of Fractogel EMD Epoxy:

1. Measure 1 g of Fractogel EMD Epoxy (size 40-90 μm, Merck KGaA, Darmstadt, Germany, catalog number 1.16961.0010, available in the UK from VWR International). Place beads in 50 ml conical tube.
2. Resuspend in 40 ml dH2O (Fractogel swells to about 4 ml per 1 g), centrifuge at 32 x *g*, 1 min in benchtop swinging bucket centrifuge, without brake.
3. Remove supernatant with pipet, resuspend in 50 ml dH2O, centrifuge again.
4. Remove supernatant with pipet, resuspend in 50 ml dH2O.
5. Incubate at 65°C 18-20 hr with gentle mixing, then centrifuge.
6. Remove supernatant with pipet, resuspend in 50 ml dH2O, centrifuge again.
7. Remove supernatant with pipet, resuspend in 50 ml dH2O, centrifuge again.
8. Remove supernatant with pipet.

Note: Preparation of IgG and coupling are largely derived from protocols from the laboratory of Michael Rout at Rockefeller University, used for coupling IgG to Epoxy Dynabeads (Oeffinger et al. (2007) Comprehensive analysis of diverse ribonucleoprotein complexes. Nat Methods. 4:951-956)

Preparation of IgG:

9. For each 1 g of Fractogel (i.e. 4 ml bed volume), dissolve 14 mg rabbit IgG (Sigma, catalog number I5006) in 1 ml 50 mM sodium phosphate pH 7.5.

10. Dialyze twice against 100 volumes of 50 mM sodium phosphate pH 7.5, 2 hours each time.
11. If there is extra IgG, aliquot and freeze in LN2 for future use.
12. (Thaw aliquot if necessary.) Centrifuge at 12,000 x *g*, 15 minutes, in refrigerated microcentrifuge to pellet/remove any aggregates.
13. Add 1 ml IgG to 7 ml of 78.5 mM sodium phosphate, pH 7.5.
14. Slowly add 4 ml of 3 M ammonium sulfate, with gentle mixing, to yield IgG solution in 50 mM sodium phosphate, 1 M ammonium sulfate. Some IgG will precipitate.
15. Filter through 0.22 mm syringe filter (the full 12 ml is typically not recovered, and typically about 30% of the IgG is lost as precipitate).

Coupling and washes:

16. Take partially deactivated Fractogel EMD Epoxy and resuspend in 50 ml 50 mM NaPO4, 1 M ammonium sulfate. Centrifuge at 32 x *g*, 1 min in benchtop swinging bucket centrifuge, without brake.
17. Remove supernatant with pipet, resuspend in 50 ml 50 mM sodium phosphate, 1 M ammonium sulfate, and centrifuge again.
18. Remove as much supernatant as possible with pipet.
19. Add filtered IgG solution to beads.
20. Transfer to 15 ml conical tube and incubate with gentle mixing/rotation 2.5-3 hr, 30°C (the absence of "dead space" in the 15 ml tube may minimize aeration/denaturation of the IgG).

    Note: After coupling, rapid washes of the beads are done in a column, but initial and final washes are done by brief centrifugation, to ensure removal of any colloidal protein aggregates that might otherwise be trapped in the interstices between beads.

21. Centrifuge the coupling reaction, 32 x *g*, 1 min, in benchtop swinging bucket centrifuge, without brake.
22. Remove supernatant with pipet, resuspend in PBS, transfer to 50 ml conical tube, add more PBS to final 50 ml volume, and centrifuge again.
23. Remove supernatant with pipet, resuspend in 50 ml PBS, centrifuge again.
24. Remove supernatant with pipet, resuspend in 25 ml PBS and transfer bead slurry to 20 ml disposable plastic column (Sigma C2353) mounted on a ring stand.
25. Suck liquid through beads by temporarily holding a 1 ml pipet tip, attached to an aspirator, at the outlet of the column. This can be done quickly, as the beads can withstand high flow rates. Avoid "drying out" the beads.
26. Plug the bottom of the column. Add 25 ml 100 mM glycine HCl, pH 2.5. Quickly cap the column and invert to mix. Immediately return to ring stand and suck liquid through the bed as before (all of this should take less than 1 min).
27. Immediately add 30 ml 10 mM Tris pH 8.8 and quickly suck liquid through as before.
28. Add 30 ml 10 mM Tris pH 8.8 again, plug and cap the column, and invert to mix. Suck liquid through the column as before.
29. Plug the bottom of the column. Add 25 ml **freshly prepared** 100 mM Triethylamine. Quickly cap the column and invert to mix. Immediately return to stand and suck liquid through the bed as before (all of this should take less than a minute).
30. Immediately add 30 ml PBS and quickly suck liquid through as before.
31. Add 30 ml PBS again, plug and cap the column, and invert to mix. Suck liquid through the column as before.

32. Resuspend the beads in 50 ml PBS. Wash on rotator 5 minutes. Centrifuge 32 x *g,* 1 min, in benchtop swinging bucket centrifuge, without brake. Remove supernatant. Repeat this wash step 3 additional times.
33. Resuspend the beads in 50 ml PBS + 0.5% Triton X-100. Wash on rotator 5 minutes. Centrifuge 32 x *g,* 1 min, without brake. Remove supernatant.
34. Resuspend beads in 50 ml PBS + 0.5% Triton X-100. Wash on rotator 15 minutes. Centrifuge 32 x *g,* 1 min, without brake. Remove supernatant.
35. Wash beads 2 more times in PBS as above and store as 20% (v/v) slurry in PBS + 0.02% NaN3.

Assaying coupling:

36. Put 25 ml of the coupled 20% slurry (i.e. 5 ml of settled beads) into each of two microcentrifuge tubes, centrifuge 12,000 x g 30 sec, and remove supernatant.
37. Resuspend one of the samples in 0.5 ml Laemmli SDS-PAGE sample buffer without bromphenol blue or reducing agents (i.e. 2% SDS, 60 mM Tris pH 6.8, 10% glycerol) and incubate at 95°C for 5 min, centrifuge to pellet beads and remove supernatant. Do nothing to the other sample.
38. Resuspend both samples in 1 ml PBS, pellet in microcentrifuge and remove supernatant. Repeat this a second time, carefully removing all liquid after the second centrifugation.
39. Add 1 ml BCA protein assay reagent to both samples, and also prepare additional controls and standards in 1 ml BCA reagent (e.g. BCA reagent alone, and 2, 4, 8 and 16 μg IgG).
40. Incubate microcentrifuge tubes at 65°C 15 min with gentle mixing to keep beads from settling (we use Eppendorf Thermomixer, set to intermittent mixing). Chill on ice after incubating to slow down the color-development reaction. (Alternatively, incubations can be done at lower temperature for longer times.)
41. Centrifuge the bead samples in microcentrifuge and recover the supernatant.
42. Read OD_{562} of samples and the standards, using BCA reagent alone as blank. Calculate the extent of coupling, based on the standards and the volume of the beads assayed. Comparison of IgG associated with beads with and without boiling in Laemmli SDS-PAGE sample buffer gives an indication of how much IgG is truly covalently bound.

    Note: The degree of coupling of IgG to Fractogel can be determined more precisely by using unreacted Fractogel EMD Epoxy beads as the "blank" in the BCA protein assay and spectrophotometry. (Appropriate beads can be obtained by setting aside a small amount of beads after step 8 and storing them at 4°C.) The unreacted Fractogel can be treated in the same way as the IgG-Fractogel samples in steps 36-41, and the BCA supernatant from this "no-IgG Fractogel" is used instead of BCA reagent alone as the "blank" in the spectrophotometer.
